# Safety of Using Traditional Chinese Medicine Injections in Primary Medical Institutions: Based on the Spontaneous Reporting System 2016–2020 in Henan Province, China

**DOI:** 10.3389/fphar.2022.761097

**Published:** 2022-04-12

**Authors:** Ziqi Yan, Zhanchun Feng, Zhiming Jiao, Ganyi Wang, Chaoyi Chen, Da Feng

**Affiliations:** ^1^ School of Medicine and Health Management, Tongji Medical College, Huazhong University of Science and Technology, Wuhan, China; ^2^ Medical Products Administration & Center for ADR Monitoring of Henan, Zhengzhou, China; ^3^ College of Public Administration, Huazhong University of Science and Technology, Wuhan, China; ^4^ School of Pharmacy, Tongji Medical College, Huazhong University of Science and Technology, Wuhan, China

**Keywords:** TCM injections, ADRs, primary medical institutions, Medication behavior, essential medicines

## Abstract

**Objective:** Traditional Chinese medicine (TCM) injection is widely used, but its adverse drug reaction (ADR) may be a serious public health concern in primary medical institutions. This research will explore the safety of TCM injections and provide clinical recommendations at the primary medical institutions.

**Method:** ADR data were collected by the Henan Adverse Drug Reaction Monitoring Center from 2016 to 2020 were analized Descriptive statistics, chi-square analysis, binary logistic regression, and Mantel-haenszel hierarchical analysis were used to identify the risk factors associated with the rational use of TCM injections in primary medical institutions.

**Results:** A total of 30,839 cases were collected in this study, 4905 cases (15.90%) were SADRs. Patients using TCM injections in primary medical institutions were more likely to cause SADRs (OR = 1.149, 95% CI: 1.061–1.245). Aged over 60 years (OR = 1.105, 95% CI: 1.007–1.212), non-essential drugs (OR = 1.292, 95% CI: 1.173–1.424), autumn (OR = 1.194, 95% CI: 1.075–1.326) and TCM injections with safflower (OR = 1.402, 95% CI: 1.152–1.706), danshen (OR = 1.456, 95% CI: 1.068–1.984) and medication reasons with chemotherapy (OR = 2.523, 95% CI: 1.182–5.386) and hypertension (OR = 1.495, 95% CI: 1.001–2.233) were more likely to suffer SADR in primary medical institutions.

**Conclusion:** In general, the number of reported cases of TCM injection was declining over time, but the proportion of SADRs in primary medical institutions increased. In the future, it is necessary to continue to restrict TCM injections at the macro policy level, and vigorously promote the varieties in the essential drug list. At the micro level, it is necessary to intervene in specific populations, specific diseases and specific drugs, first start with them, step by step, and effectively prevent SADR occurrences in primary medical institutions.

## Introduction

Chinese medicine has a history of thousands of years in China. More than 9,000 Chinese medicine preparations have been approved for use in China (Zhu et al., 2010). Traditional Chinese medicine (TCM) injections have a history of 80 years and are still widely used today (Jing-Yao et al., 2016). Compared with other dosage forms, such as tablets, pills, and oral liquid, they have the advantage of rapid onset (Wang et al., 2017). In 2017, China approved the sale of 134 kinds of TCM injections from 224 manufacturers (Li et al., 2018). Sales of TCM injections have reached >30 billion RMB and account for one-third of all TCM sales in hospitals (Li et al., 2018).

However, with the application of TCM injections, an increasing number of adverse drug reactions (ADRs) have been reported (Li et al., 2019). Such findings have raised concerns about the safety and potential toxicity of currently used TCM injections. In response to this situation, the National Food and Drug Administration (CFDA) has adopted a series of measures. For example, although many kinds of TCM injections exist, only 10 were included in the National Essential Drugs List (2018)1^1^. Since 2017, the National Medical Insurance Catalogue has restricted the use of Shuanghuanglian, Reduning, Qingkailing, Ciwujia, Xuesaitong, and many more commonly used TCM injections^2^ ^,^ ^3^ ^,^ ^4^. They can only be used in secondary and above medical institutions and are restricted to certain populations and diseases. Given such management measures, in 2020, about 74,800 ADRs were caused by TCM injections, which was a decrease of 14.5% from 87,500 in 2019. The proportion of injection in the ADRs of TCM also decreased significantly.^5^ ^,^ ^6^ However, as a result of inadequate supervision and insufficient patient awareness, many primary medical institutions still use TCM injections. Most of these medical institutions are located in the suburbs and rural areas of China, and effective response is difficult if ADRs occur. Nevertheless, safety studies on TCM injections in primary medical institutions remain sparse.

Spontaneous reporting system (SRS) has the advantage of covering a large number of patients and a wide range of drugs, and nearly 16.87 million ADR reports have been collected from 1999 to 2020 (Guo et al., 2015). Different from the current research, this article started from the perspective of management by focusing on a variety of TCM injections in primary medical institutions about safety by analyzing China’s provincial SRS database.

## Methods

### Data Collection

The data of adverse drug reaction reports collected by Adverse Drug Reaction Monitoring Center’s SRS of Henan Province from January 2016 to December 2020 were classified, analyzed, and spontaneously reported by medical institutions, enterprises, and the public in Henan.

The data were cleaned and preprocessed to ensure that they were clean and complete. The ADR database includes all reported ADRs. Reports of TCM injections with the registered category of Chinese medicine and the drug approval number containing “z” in the NMPA were selected for inclusion (Huang et al., 2021). On the basis of the Provisions of Drug Registration issued by NMPA, the drug approval number initials represent different types of drugs, in which “z” means TCM.^7^ A total of 30,389 reports of ADRs caused by TCM injections were included in the analysis.

### Data Setting

This study divided the data set into the following parts: 1. sample characteristics, including gender, age, ADR history, smoking or drinking history, and whether suffering from multiple diseases or polypharmacy; 2. medication factors, including year, season, method, and type of institution of medication, and essential drug history; 3. injection characteristics via text analysis to summarize the top 15 TCM injections with the most ADRs; and 4. medication characteristics via text analysis to summarize the top 10 reasons for medication with the most ADRs.

In terms of medical institutions, this study defines institutions that provide basic public health services and basic medical services as primary medical institutions, whereas other comprehensive medical institutions are non-primary medical institutions in accordance with relevant national policies.

This study was based on the regulations of the National Adverse Drug Reaction Monitoring Center. Among the reported ADRs, death; teratogenic, carcinogenic, or birth defect; permanent sequelae; permanent damage to organ function; and hospitalization or prolonged hospital stay were regarded as “serious ADRs” (Abbreviated as SADRs). Other cases were regarded as “normal ADRs.”

### Statistical Analysis

The descriptive statistical methods were used to summarize data on patient’s demographic characteristics, TCM injections, and reasons for medications. Data were summarized as frequencies (*n*) and percentages (%) for categorical variables. The chi-square test was used for univariate analysis of the SADRs. Binary logistic regression analyses were employed to identify the potential factors and vulnerable populations related to SADRs. The demographic variables, types and reasons for personal medication, and medication method were set as independent variables, and severity of ADRs was set as the outcome variables (normal and serious). To further identify the risk factors that cause SADRs when using TCM injections in primary medical institutions, Mantel–Haenszel hierarchical analysis of medical Institution Level factors was conducted across sub-characteristics. Unstandardized regression coefficients (*β*) and odd ratios (ORs) and their 95% confidence intervals (95% CIs) were used to quantify the associations between variables and misconceptions regarding SADRs. Data analyses were performed using Statistical Package for the Social Sciences (SPSS) software, version 23.0. The value of *p* < 0.05 was considered statistically significant (Zhou et al., 2020).

## Results

### Variation Characteristics

**FIGURE 1:**
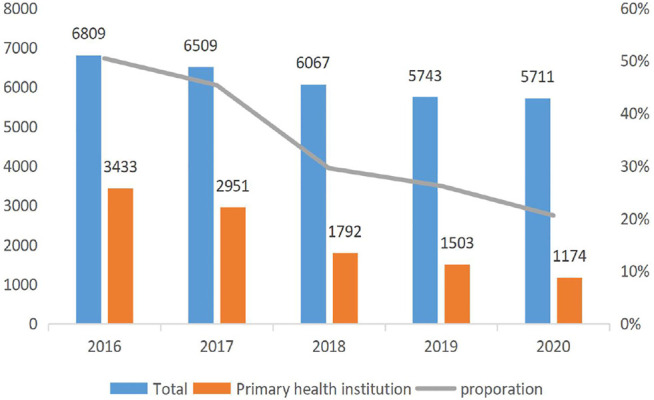
Number of reports in each year.

**FIGURE 2:**
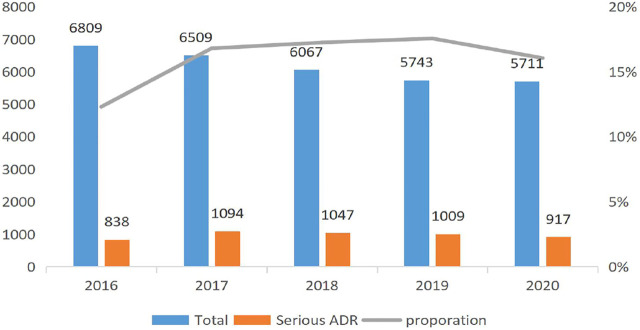
Severity report in each year.

**FIGURE 3:**
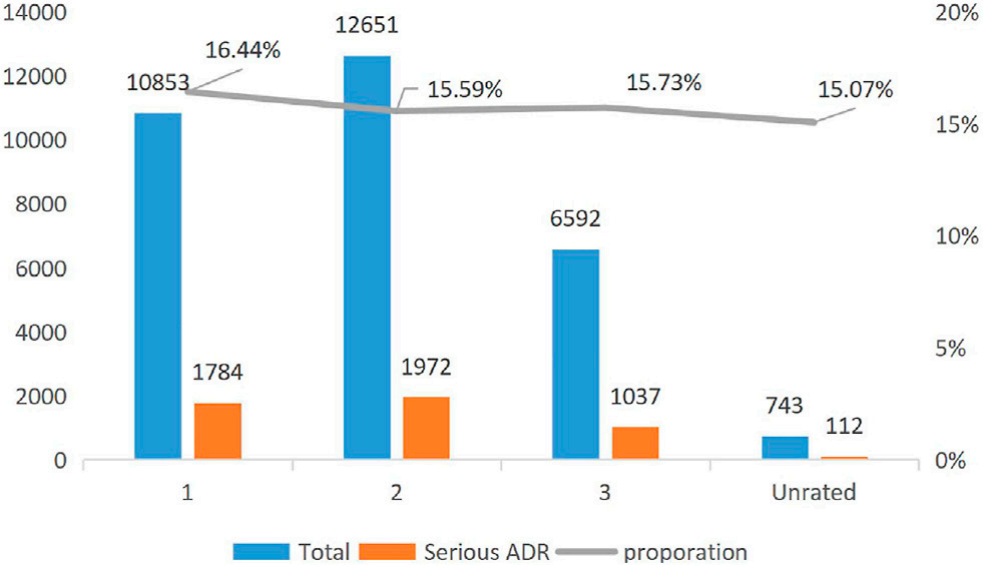
Severity report in different levels From 2016 to 2021.

Figure 1 reports the trend of the ADRs of TCM injection from 2016 to 2020. The number of cases reported each year and the proportion of reports from the primary medical institutions decreased. Figure 2 describes the trend of SADRs, and the proportion was higher than the national average of 10%. Figure 3 shows the proportion of SADRs in different levels of medical institutions. The proportion of primary medical institutions was found to be the highest.

### The Sample Characteristics

A total of 30,839 cases were collected in this study, of which 4905 cases (15.90%) were SADRs. The other 25,934 cases were normal ADRs. The average age of the participants was 54.16 years. Approximately 47.22% of them were men, and the elderly (>60) accounted for 46.25% of the total samples. Besides, 2.88% of patients had a history of ADRs, and 13.03% suffered from more than one disease before the ADR occurred. A small number of patients had a family history of ADRs and multi-drug behavior. The detailed demographic characteristics are shown in Table 1.

**TABLE 1:** Number and proportion of serious and non-serious reports by personal characteristics.

Characteristics	Serious N (%)	Non-serious N (%)	Total	*p-Value*
Age				
Below 18	318 (12.59)	2208 (87.41)	2526	˂0.001
18–44	362 (15.03)	2046 (84.97)	2408	
45–60	1856 (16.02)	9727 (83.98)	11,583	
Above 60	2312 (16.21)	11,952 (83.79)	14,264	
Gender				
Male	2412 (16.56)	12,151 (83.44)	14,563	0.003
Female	2487 (15.31)	13,752 (84.69)	16,239	
Personal ADR past history				
Yes	158 (17.79)	730 (82.21)	888	0.003
No	3409 (16.26)	17,552 (83.74)	20,961	
Unknown	1338 (14.88)	7652 (85.12)	8,990	
Family ADR past history				
Yes	5 (45.45)	6 (54.55)	11	0.001
No	2907 (16.09)	15,164 (83.91)	18,071	
Unknown	1992 (15.61)	10,765 (84.39)	12,757	
Drinking or Smoking				
Yes	514 (19.02)	2188 (80.98)	2,702	˂0.001
No	4391 (15.61)	23,746 (84.39)	28,137	
Polypharmacy				
Yes	17 (22.67)	58 (77.33)	75	0.109
No	4888 (15.89)	25,876 (84.11)	30,764	
Original disease				
1	4097 (15.27)	22,725 (84.73)	26,822	˂0.001
≥2	808 (20.11)	3209 (79.89)	4,017	

### Injection Characteristics

Table 2 shows the number and proportion of serious and non-serious reports by TCM injections (top 15 ordered by the number of ADR reports and the ADRs caused by these 15 TCM injections accounted for more than 75% of the entire database). Differences in the proportion of SADRs were found (*p* < 0.001). Among them, Qingkailing injection had the most ADR reports, whereas Huangqi injection had highest proportion of SADRs.

**TABLE 2:** Number and proportion of serious and non-serious reports by TCM injections.

TCM injections	Serious N (%)	Non-serious N (%)	Total	*p-Value*
Qingkailing injection	583 (14.15)	3538 (85.85)	4,121	<0.001
Xuesaitong injection	416 (15.31)	2302 (84.69)	2,718	
Safflower injection	491 (18.61)	2148 (81.39)	2,639	
Shenmai injection	379 (18.63)	1655 (81.37)	2,034	
Mailuoning injection	301 (18.18)	1355 (81.82)	1,656	
Shuanghuanglian injection	240 (16.25)	1237 (83.75)	1,477	
Xueshuantong injection	249 (19.70)	1015 (80.30)	1,264	
Danshen injection	191 (15.19)	1066 (84.81)	1,257	
Reduning injection	158 (13.39)	1022 (86.61)	1,180	
Shuxuening injection	158 (14.03)	968 (85.97)	1,126	
Xiangdan injection	161 (16.16)	835 (83.84)	996	
Astragalus injection	198 (21.09)	741 (78.91)	939	
Breviscapine injection	105 (14.40)	624 (85.60)	729	
Ciwujia injection	92 (14.44)	545 (85.56)	637	
Tanreqing injection	68 (11.39)	529 (88.61)	597	
Others	1115 (14.93)	6354 (85.07)	7,469	

### Medication Characteristics

Table 3 shows the number and proportion of serious and non-serious reports based on the reasons for medication (top 10 ordered by the number of ADR reports and the ADRs caused by these 10 reasons accounted for more than 75% of the entire database). Among them, “improve body circulation” had the most ADR reports, whereas “medication for hypertension” had the highest proportion of SADRs.

**TABLE 3:** Number and proportion of serious and non-serious reports by Reason for medication.

Reason for medication	Serious N (%)	Non-serious N (%)	Total	*p-Value*
Improve body circulation	837 (16.71)	4172 (83.29)	5,009	0.170
Upper respiratory tract infection	608 (14.94)	3461 (85.06)	4,069	
Coronary Heart disease	446 (16.96)	2183 (83.04)	2,629	
Other brain diseases	427 (16.25)	2200 (83.75)	2,627	
Cerebral infarction	409 (15.88)	2167 (84.12)	2,576	
Bringing down a fever	289 (14.24)	1741 (85.76)	2,030	
Chemotherapy	198 (15.09)	1114 (84.91)	1,312	
Other heart diseases	202 (16.07)	1055 (83.93)	1,257	
Lower respiratory tract infection	173 (16.80)	857 (83.20)	1,030	
Hypertension	126 (17.03)	614 (82.97)	740	
Others	1189 (15.74)	6366 (84.26)	7,555	

### Frequently Reported ADRs

Table 4 shows the top 10 the types of ADRs that occurred in normal and SADR patients. A total of 25,934 patients with normal ADRs developed 39,090 adverse reaction symptoms. These symptoms were concentrated in skin reactions, such as rash (14.63%) and pruritus (13.89%). Approximately 5,934 patients with SADRs developed 11,492 adverse reaction symptoms. Most of them were dyspnea (11.57) and chest tightness (11.41). The top 10 adverse reaction symptoms that included adverse reactions affecting the circulatory system and the whole body, such as cardiopalm and anaphylactic shock, also had a serious impact on prognosis.

**TABLE 4:** Number and proportion of ADRs and SADRs (Top 10).

Rank	ADR (N = 39,090)	*n*	% (n/N)	Rank	SADR (*n* = 11,492)	*n*	% (n/N)
1	Rash	5,720	14.63	1	Dyspnoea	1,330	11.57
2	Pruritus	5,428	13.89	2	Chest distress	1,311	11.41
3	Chest distress	2,985	7.64	3	Cold shiver	928	8.08
4	Nausea	2,756	7.05	4	Fever	895	7.79
5	Dizzy	1,993	5.10	5	Cardiopalm	862	7.50
6	Emesia	1,360	3.48	6	Pruritus	638	5.55
7	Allergy	1,385	3.54	7	Rash	625	5.44
8	Fever	1,175	3.01	8	Nausea	305	2.65
9	erubescence	1,141	2.92	9	Anaphylactic shock	299	2.60
10	Headache	859	2.20	10	Allergy	261	2.27

### Medication Factors That Affect the Degree of ADRs

Table 5 analyzes the influence of medication factors on the SADRs with the inclusion of variables in Tables 1–3. The results showed that season, injection method, and medical institution were related to SADRs. Among them, we focused on the patients who used TCM injections in primary medical institutions (vs. non-primary medical institutions, *β*: 0.139).

**TABLE 5:** Severity level of TCM injections by demographic variables and medications.

Medications	Risk of serious adverse reactions		*p-Value*
	B	Or (95%CI)	
Years (refer to 2016)			
2017	0.396	1.486 (1.346–1.640)	<0.001
2018	0.484	1.623 (1.466–1.796)	<0.001
2019	0.494	1.639 (1.478–1.817)	<0.001
2020	0.409	1.505 (1.353–1.675)	<0.001
Medication season(refer to spring)			
Summer	0.041	1.042 (0.956–1.135)	0.348
Autumn	0.213	1.237 (1.139–1.344)	<0.001
Winter	0.042	1.043 (0.935–1.164)	0.447
Essential drugs(refer to yes)			
No	−0.108	0.898 (0.766–1.053)	0.185
Intravenous drip(refer to no)			
Yes	0.284	1.328 (1.130–1.562)	0.001
Primary medical institutions(refer to no)			
Yes	0.139	1.149 (1.061–1.245)	0.001

Note: OR, odds ratio; CI, confidence interval; *p˂0.05, **p˂0.01.All the variables in Table 1, 2, and 3 have been included.

### Factors That Cause SADRs in Primary Medical Institutions

Tables 6, 7 analyze the sample characteristics, medication methods, TCM injections, and medication reasons at different levels. The results of stratified analysis showed that the population aged over 60 years (OR = 1.105, 95% CI: 1.007–1.212), non-essential drugs (OR = 1.292, 95% CI: 1.173–1.424), autumn (OR = 1.194, 95% CI: 1.075–1.326), TCM injections with safflower (OR = 1.402, 95% CI: 1.152–1.706) and danshen (OR = 1.456, 95% CI: 1.068–1.984), and medication reasons with chemotherapy (OR = 2.523, 95% CI: 1.182–5.386) and hypertension (OR = 1.495, 95% CI: 1.001–2.233) were more likely to suffer SADR in primary medical institutions than their counterparts.

**TABLE 6:** Hierarchical analysis of the personal and medication characteristics.

Variables	Primary medical institutions	Non-primary medical institutions	OR (95% CI)
	Serious/%	Serious/%	
Personal characteristics			
Age			
Below 18	76/694 (10.95)	242/1832 (13.21)	0.808 (0.614–1.063)
18–44	135/882 (15.31)	227/1526 (14.88)	1.034 (0.821–1.303)
45–60	711/4353 (16.33)	1145/7230 (15.84)	1.037 (0.937–1.149)
Above 60	838/4899 (17.11)	1474/9365 (15.74)	1.105 (1.007–1.212)*
Gender			
Male	853/4982 (17.12)	1559/9581 (16.27)	1.063 (0.970–1.165)
Female	928/5856 (15.85)	1559/10,383 (15.01)	1.066 (0.976–1.164)
Personal ADR past history			
Yes	16/137 (11.68)	142/751 (18.91)	0.567 (0.326–0.985) *
No	1232/6991 (17.62)	2177/13,970 (15.58)	1.159 (1.073–1.251)**
Unknown	536/3725 (14.39)	802/5265 (15.23)	0.935 (0.831–1.053)
Family ADR past history			
Yes	1/2 (50.00)	5/9 (55.56)	0.800 (0.037–17.196)
No	1064/6185 (17.20)	1843/11,886 (15.51)	1.132 (1.042–1.230)**
Unknown	719/4666 (15.41)	1273/8091 (15.73)	0.976 (0.883–1.078)
Drinking or Smoking			
Yes	258/1280 (20.15)	256/1422 (18.00)	1.150 (0.949–1.394)
No	1526/9573 (15.94)	2865/18,564 (15.43)	1.039 (0.971–1.112)
Polypharmacy			
Yes	2/4 (50.00)	15/71 (21.13)	3.733 (0.485–28.744)
No	1782/10,849 (16.43)	3106/19,915 (15.60)	1.064 (0.998–1.133)
Original disease			
1	1638/10,233 (15.71)	2459/16,589 (14.82)	1.095 (1.023–1.172)**
≥2	146/620 (23.55)	662/3397 (19.49)	1.273 (1.038–1.561)*
Medication characteristics			
Essential drugs			
Yes	1108/7163 (15.47)	1286/7577 (16.97)	0.895 (0.820–0.977)*
No	676/3690 (18.32)	1835/12,409 (14.79)	1.292 (1.173–1.424)**
Intravenous drip			
Yes	1686/10,134	3034/19,240	1.066 (0.999–1.138)
No	98/719	87/746	1.195 (0.878–1.628)
Medication season			
Spring	368/2630	772/5033	0.898 (0.785–1.027)
Summer	499/3241	923/5967	0.995 (0.883–1.120)
Autumn	741/3920	1006/6159	1.194 (1.075–1.326)**
Winter	176/1062	420/2827	1.138 (0.939–1.380)

Note: OR, odds ratio; CI, confidence interval; *p ˂ 0.05, **p ˂ 0.01.

**TABLE 7:** Hierarchical analysis of the Types of drugs and reasons for medication.

Variables	Primary medical institutions	Nonprimary medical institutions	OR (95% CI)
	Serious/%	Serious/%	
Used TCM injections			
Qingkailing injection	159/1103 (14.4)	424/3018 (14.0)	0.970 (0.797–1.182)
Xuesaitong injection	116/797 (14.6)	300/1921 (15.6)	0.920 (0.730–1.161)
Safflower injection	258/1206 (21.4)	233/1433 (16.3)	1.402 (1.152–1.706)**
Shenmai injection	108/569 (19.0)	271/1465 (18.5)	1.032 (0.806–1.322)
Mailuoning injection	166/918 (18.1)	135/738 (18.3)	0.986 (0.767–1.267)
Shuanghuanglian injection	121/840 (14.4)	119/637 (18.7)	0.733 (0.555–0.966)*
Xueshuantong injection	32/158 (20.3)	217/1106 (19.6)	1.040 (0.687–1.576)
Danshen injection	91/501 (18.2)	100/756 (13.2)	1.456 (1.068–1.984)*
Reduning injection	15/72 (20.8)	143/1108 (12.9)	1.776 (0.979–3.221)
Shuxuening injection	39/236 (16.5)	119/890 (13.4)	1.283 (0.865–1.902)
Xiangdan injection	90/575 (15.7)	71/421 (16.9)	0.915 (0.651–1.285)
Astragalus injection	116/493 (23.5)	82/446 (18.4)	1.366 (0.994–1.876)
Breviscapine injection	45/321 (14.0)	60/408 (14.7)	0.946 (0.623–1.436)
Ciwujia injection	8/124 (6.5)	84/513 (16.4)	0.352 (0.166–0.748)**
Tanreqing injection	2/19 (10.5)	66/578 (11.4)	0.913 (0.206–4.039)
Reason for medication			
Improve body circulation	109/586 (18.60)	728/4423 (16.46)	1.160 (0.928–1.449)
Upper respiratory tract infection	364/2507 (14.52)	244/1562 (15.62)	0.917 (0.769–1.094)
Coronary Heart disease	227/1255 (18.09)	219/1374 (15.94)	1.165 (0.950–1.428)
Other brain diseases	238/1516 (15.70)	189/1111 (16.74)	0.908 (0.737–1.120)
Cerebral infarction	189/1084 (17.44)	220/1492 (14.75)	1.221 (0.987–1.010)
Bringing down a fever	95/713 (13.32)	194/1317 (14.73)	0.890 (0.683–1.159)
Chemotherapy	10/33 (30.30)	188/1279 (14.70)	2.523 (1.182–5.386)*
Other heart diseases	92/535 (17.20)	110/722 (15.24)	1.155 (0.854–1.564)
Lower respiratory tract infection	84/444 (18.92)	89/586 (15.19)	1.303 (0.939–1.808)
Hypertension	83/429 (19.35)	43/311 (13.83)	1.495 (1.001–2.233)*

Note: OR, odds ratio; CI, confidence interval; *p ˂ 0.05, **p ˂ 0.01.

## Discussion

Statistics show that the reports of TCM injections reported by the SRS have shown a downward trend in the past 5 years, and the proportion of primary medical institutions is declining yearly. We believe that this decline is due to the introduction of an increasing number of restrictive measures for TCM injections in recent years, especially the strict management and restriction of the use of TCM injections at primary medical institutions (Xiao et al., 2013).

However, given the lack of clinical evidence or experience on the safety and effectiveness of TCM injections, and most TCM injections have been approved for sale many years ago, by today’s standards, there is insufficient evidence of safety and effectiveness (Liu et al., 2016). The results of this study showed that 15.9% of patients had ADRs after using TCM injections, which was higher than the average level of 9.1% in Henan Province in our research database.

Data from this study further suggested that SADR patients have more SADRs, such as dyspnea, chest distress, cardiopalm, and anaphylactic shock, are common in this population. Studies have shown that these adverse reaction symptoms cause poor prognosis (Bouzillé et al., 2018; Li et al., 2019).

Compared with non-primary medical institutions, the SADRs faced by primary medical institutions are more serious because of the lack of professionals or equipment and infrequent related training, so the risk of using Chinese medicine injections in primary medical institutions is high (Faria et al., 2014). Thus, primary medical institutions still cannot relax the supervision of TCM injections.

In our cases, the risk of SADRs among the elderly (>60) injected with TCM at primary medical institutions significantly increased. This result was speculated to be correlated with the patients’ own constitution, metabolism, and decline of organs (Wang et al., 2018). The safety of elderly medication must be given close attention (Qing et al., 2019).

Using TCM injections outside the essential medicine list in primary medical institutions could also lead to poor results. Interestingly, the use of injections in the essential medicine list has a low probability of SADR, thereby illustrating the importance of popularizing essential medicines. Season was also a factor that should be paid attention. Previous studies on ADRs and seasons reported that the proportions of SADRs in autumn and winter increase significantly because patients are highly sensitive to drug reactions during this time. Weather, flu, and seasonal allergies also yielded similar results (Chabanon et al., 2021; Deb et al., 2021). However, the reasons related to TCM injections need to be further studied.

Shuanghuanglian and Ciwujia injection have low risk of SADRs when used in primary medical institutions. However, they can also cause allergic shock, so China also restricts their use. Safflower and danshen injections are more risky when used in primary medical institutions. Safflower injection can cause allergic shock, and danshen injection is a potential vascular toxic drug in high dosage, so it should not be used far beyond its recommended dosage for clinical treatment (Sun et al., 2013; Wang et al., 2015). Given that Chinese medicine is considered only a complementary treatment for the control of hypertension and protection of target organs (Wu and Dong, 2015), if patients want to stabilize their condition for a long time, then systematic treatment in a comprehensive medical institution is necessary. Chemotherapy is also not suitable for the use of TCM injections, because the patients need to be treated in professional medical institutions; given their poor physique and health, injection at primary medical institutions will only increase their risk of SADRs (Zhang et al., 2014).

In summary, the innovation of this study was the analysis of the harm of using TCM injection in different levels of medical institutions and proposal of recommendations to avoid serious consequences. Nevertheless, this study had potential limitations. First, the effect estimated in the study was based on the data only in Henan Province. Although the data were considerable, the external validity of the conclusion still needs to be improved. Second, some recorded ADR information was incomplete, and some indicators had a few missing values, which may cause a certain degree of bias. Finally, given the insufficient content of the original database, this study did not specifically analyze the relationship between ADRs and drugs used.

## Conclusion

In general, the number of reported cases of TCM injections is declining, which is important to achieve safe medication. However, the proportion of SADRs in primary medical institutions has increased, and the regression results also showed that the level of drug administration institutions was a risk factor. Although the government has introduced many regulatory policies, banning primary medical institutions from using TCM injections in the near future was unrealistic. Therefore, this study demonstrated that populations (>60), drugs (safflower and danshen), and reason for medication (chemotherapy and hypertension) need to be supervised, and recommendations for the use of essential drugs must be made. These measures can help in reducing the incidence of SADRs.

In the future, restriction of TCM injections should be continued at the macro policy level, and various essential drug lists must be promoted; at the micro level, intervention is necessary in specific populations, specific diseases, and specific drugs to effectively prevent SADR occurrences in primary medical institutions. This study also proved that SADR was likely to occur when using TCM injections in primary medical institutions, and specific factors only occurred at primary medical institutions. This work reflects the necessity of the state to introduce regulatory policies and further points out the direction for policy pertinence.

## Data Availability

The data analyzed in this study is subject to the following licenses/restrictions: Data may be obtained from a third party and are not publicly available. Requests to access these datasets should be directed to Center for ADR Monitoring of Henan.

## Footnotes

1 National Essential Medicines List—2018 Edition—National Health Commission of the People’s Republic of China [EB/OL] [2021/7/30].http://www.nhc.gov.cn/wjw/jbywml/201810/600865149f4740eb8ebe729c426fb5d7.shtml.

2 The Ministry of Human Resources and Social Security Issued the National Basic Medical Insurance, Industrial Injury Insurance and Maternity Insurance Drug Catalog (2017 Edition)_Departmental Government Affairs_Chinese Government Network [EB/OL] [2021/7/30].http://www.gov.cn/xinwen/2017–02/23/content_5170392.htm.

3 Notice of the Ministry of Human Resources and Social Security of the National Medical Insurance Administration on Printing and Distributing the “National Basic Medical Insurance, Work Injury Insurance and Maternity Insurance Drug Catalog (2020)”_Medical Management_Chinese Government Network [EB/OL] [2021/7/30]. http://www.gov.cn/zhengce/zhengceku/2020-12/28/content_5574062.htm.

4 National Medical Insurance Administration Policies and Regulations Notice of the Ministry of Human Resources and Social Security of the National Medical Insurance Administration on Printing and Distributing the National Basic Medical Insurance, Work Injury Insurance and Maternity Insurance Drug Catalog [EB/OL] [2021/7/30]. https://www.nhsa.gov.cn/art/2019/8/20/art_37_1666.html.

5
https://www.cdr-adr.org.cn/tzgg_home/202004/t20200410_47300.html.

6 https://www.nmpa.gov.cn/directory/web/nmpa/xxgk/fgwj/gzwj/gzwjyp/20210325170127199.html.

7 Provisions of Drug Registration [EB/OL] [2022/2/22]. https://www.nmpa.gov.cn/xxgk/fgwj/bmgzh/20200330180501220.html.
